# Thyroid primary and metastatic malignant tumours of poor prognosis may mimic subacute thyroiditis - time to change the diagnostic criteria: case reports and a review of the literature

**DOI:** 10.1186/s12902-019-0415-y

**Published:** 2019-08-06

**Authors:** Magdalena Stasiak, Renata Michalak, Andrzej Lewinski

**Affiliations:** 1 Department of Endocrinology and Metabolic Diseases, Polish Mother’s Memorial Hospital - Research Institute, 281/289 Rzgowska St., 93-338 Lodz, Poland; 2 Department of Endocrinology and Metabolic Diseases, Medical University of Lodz, Lodz, Poland

**Keywords:** Subacute thyroiditis, Diagnostic criteria, Metastasis, Thyroid cancer, Painful goiter

## Abstract

**Background:**

The diagnosis of subacute thyroiditis (SAT) is based mainly on the presence of painful thyroid goitre and a significant increase in erythrocyte sedimentation rate (ESR). Proceeding according to these diagnostic criteria may lead to an incorrect diagnosis and treatment. Extremely dangerous is the situation when the diagnosis of SAT is erroneously made based on criteria other than ultrasound (US) image and fine needle aspiration biopsy (FNAB), which leads to delayed diagnosis of malignant tumour with poor prognosis.

**Case presentation:**

Five patients with typical SAT symptoms are presented. In all of them, anaplastic thyroid cancer or metastatic thyroid tumours were finally diagnosed as the cause of the initial symptoms resembling SAT. Most of the patients were initially misdiagnosed and the proper diagnosis of malignancy was delayed.

**Conclusions:**

The authors have proposed the new diagnostic criteria for SAT, and strongly suggest that thyroid gland US should be included in the main criteria of SAT diagnosis, together with FNAB result excluding the presence of malignant tumour.

## Background

Subacute thyroiditis (SAT), also known as de Quervain thyroiditis or granulomatous thyroiditis, is an inflammatory disease of supposed viral origin. Susceptibility to the disease is associated with the occurrence of certain types of human leukocyte antigens, which can influence the course of the disease [1]. The prevalence of SAT is the highest in middle aged women; female patients account for 75–80% of individuals with the disease [2, 3]. For several decades, diagnosis has been based on the presence of painful or tender, palpable thyroid nodule or goitre and a significant increase in erythrocyte sedimentation rate (ESR), with these two parameters being considered as the main diagnostic criteria [4, 5]. The pain typically radiates to ears, mandible and to upper part of the chest. It has been believed that both main criteria plus any two additional criteria should be met to confirm the diagnosis. Additional criteria include: significantly reduced iodine uptake (RAIU), transient thyrotoxicosis, fever, typical ultrasound (US) image, typical image of cytological material in fine needle aspiration biopsy (FNAB), and low concentration of anti-thyroid antibodies [4, 5]. However, proceeding in accordance with the above mentioned criteria may lead to an incorrect diagnosis and treatment. Recently, the presence of anti-thyroid antibodies has been described in even more than 25% of the patients, so this criterion seems to be debatable [3]. Moreover, it is worth recalling that SAT cases occurring without pain are more and more often described [6]. The basic diagnostic criterion is not met in such cases, while the disease is confirmed by the typical FNAB result with the presence of multinucleated giant cells and follicular epithelial cells against a dirty background. In such a patient, lack of pain cannot exclude SAT if other criteria confirm diagnosis. Much more dangerous, however, is the reverse situation, when both main SAT criteria and 2 additional criteria (other than FNAB) are fulfilled, and the wrong SAT diagnosis significantly delays the treatment of cancer, which may clinically mimic SAT. In this paper, we present a series of our own 5 patients and a review of the literature which indicate that the criteria for SAT diagnosis should utterly include US, and – in majority of cases – also FNAB.

## Case presentation

### Case 1

A 60-year-old man, long time smoker, presents with a one month history of marked pain and swelling of the left side of his neck. The pain radiated to the left ear and to the sternum area. In the neck, there was a hard, painful tumour, about 6 cm in diameter. The patient denied any other symptoms, including fever and dyspnoea. Three weeks earlier, the patient was diagnosed with SAT by an internal medicine specialists at an outpatient clinic. The diagnosis was based only on the neck pain and ESR up to 84 mm/h. Neither US nor other additional diagnostic procedures were performed. A treatment with a non-steroidal anti-inflammatory drug (NSAID) was started, but no improvement of the patient’s condition was achieved. Thus, the patient decided to come to the emergency room of our hospital. The laboratory results obtained in our Department are presented in Table 1.

**Table 1:** The patients` laboratory results obtained in our Department

Test [reference range and unit]	Patient 1	Patient 2	Patient 3	Patient 4	Patient 5
ESR [< 10]	77	93	94	39	38
WBC [4000–10,000/ul]	8370	4180	3680	4900	4770
CRP [< 1.0 mg/dl]	5.04	15.17	NA	NA	NA
TSH 0.27–4.2 mIU/l]	1.31	0.09	0.02	8.4	1.65
FT4 [0.93–1.7 ng/dl]	1.20	2.09	1.27	0.53	1.34
FT3 [2.6–4.4 pg/ml	3.33	2.61	3.5	2.54	2.56
anti-TPO [< 34 IU/l]	21.49	NA	NA	NA	< 5.0
anti-Tg [< 115 IU/l]	395	NA	NA	NA	13.89
TRAb [< 1.75 IU/l]	< 0.3	NA	NA	NA	NA

Abbreviations: *anti-Tg, thyroglobulin antibodies* anti-TPO, thyroid peroxidase antibodies, *CRP* C reactive protein, *ESR* erythrocyte sedimentation rate, *FT3* free triiodothyronine, *FT4* free thyroxine, *NA* not available, *TRAb* TSH receptor antibodies, *TSH* thyroid stimulating hormone, *WBC* white blood count

**Fig. 1:**
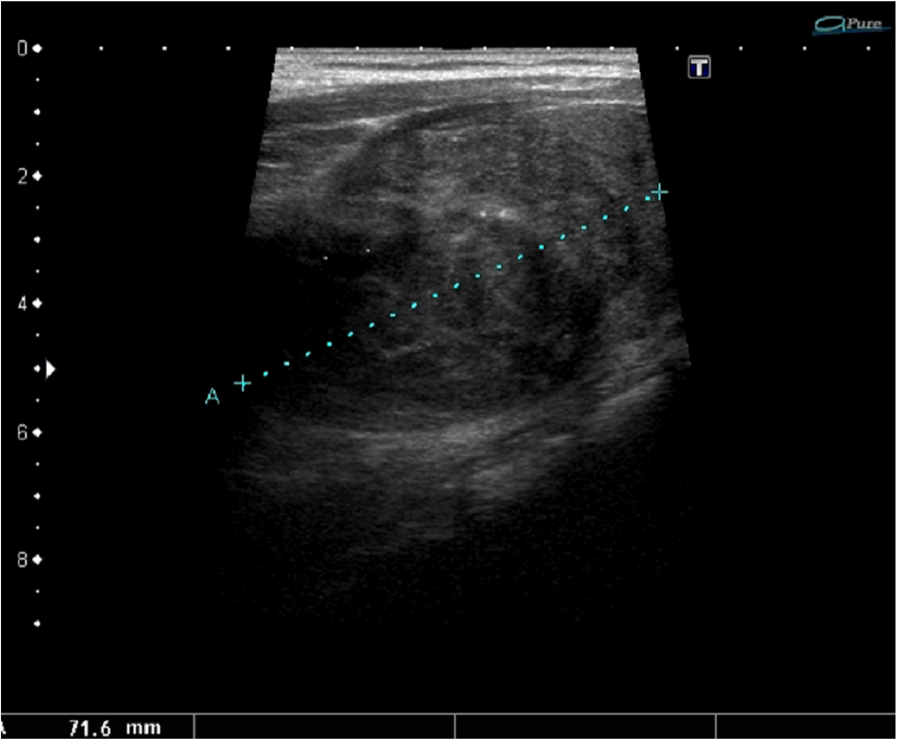
A large tumour of the thyroid left lobe, US image not typical for SAT, visible muscle infiltration, intra-tumour microcalcifications and quite clear tumour margins

**Fig. 2:**
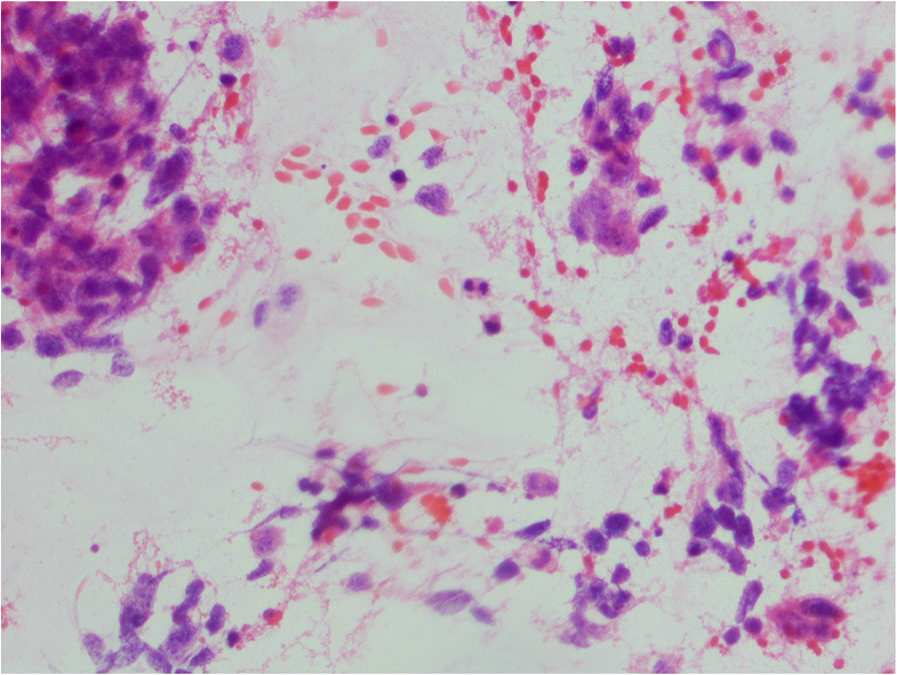
Cytology smear shows non-small cell carcinoma cells, dispersed and in groups (hematoxylin-eosin staining; light microscopy, magnification × 300)

**Fig. 3:**
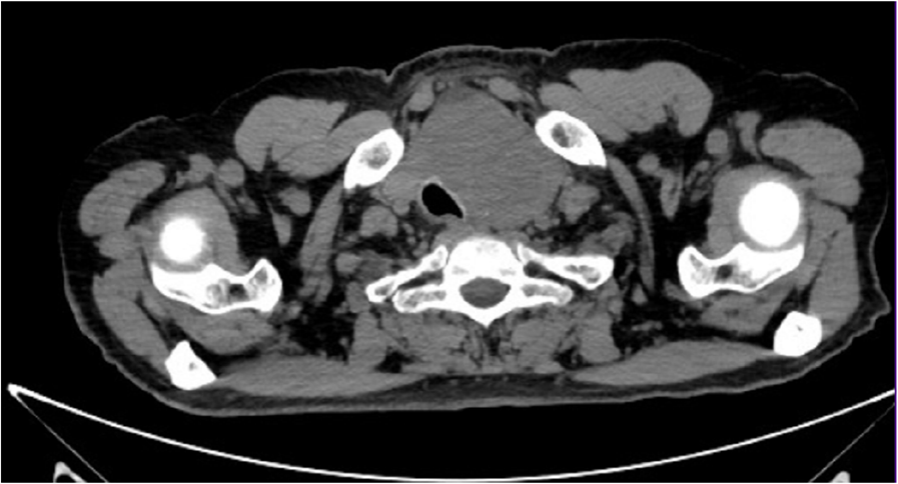
A large tumour of the thyroid, with visible compression and displacement of the trachea – metastasis of non-small cell lung cancer

Ultrasound revealed an unchanged right thyroid lobe, while the left lobe was significantly enlarged (44 × 61 × 100 mm) and almost completely filled by a heterogeneous tumour 44 × 55 × 70 mm, with numerous calcifications, almost completely without vascularisation (Fig. 1). The tumour infiltrated adjacent thyroid tissue, which could give an image similar to hypoechoic areas in the SAT, but the features of superficial neck muscle infiltration were also visible (Fig. 1). No pathological lymph node was revealed in US. Tumour FNAB revealed epithelial cells with eosinophilic cytoplasm and nuclei located at one of the cytoplasmic poles, showing distinct anizocytosis, anizocoria and heterochromasia (Fig. 2). Cytological diagnosis was non-small cell cancer. Neck and chest computed tomography (CT) revealed a large tumour of the thyroid, with visible compression and displacement of the trachea (Fig. 3), as well as numerous tumours of size up to 35 mm within both lungs (most likely metastases), without a clearly evident primary lesion. The patient was referred to the oncology clinic, where - after confirmation that the thyroid tumour is a metastasis of non-small cell lung cancer (NSCLC), the patient was qualified for palliative radiotherapy. In the meantime, the patient started suffering from dyspnoea.. The patient died 2 months after the correct diagnosis was established. Initial SAT diagnosis delayed treatment for about a month which is a dramatic waste of time in the case of NSCLC.

### Case 2

**Fig. 4:**
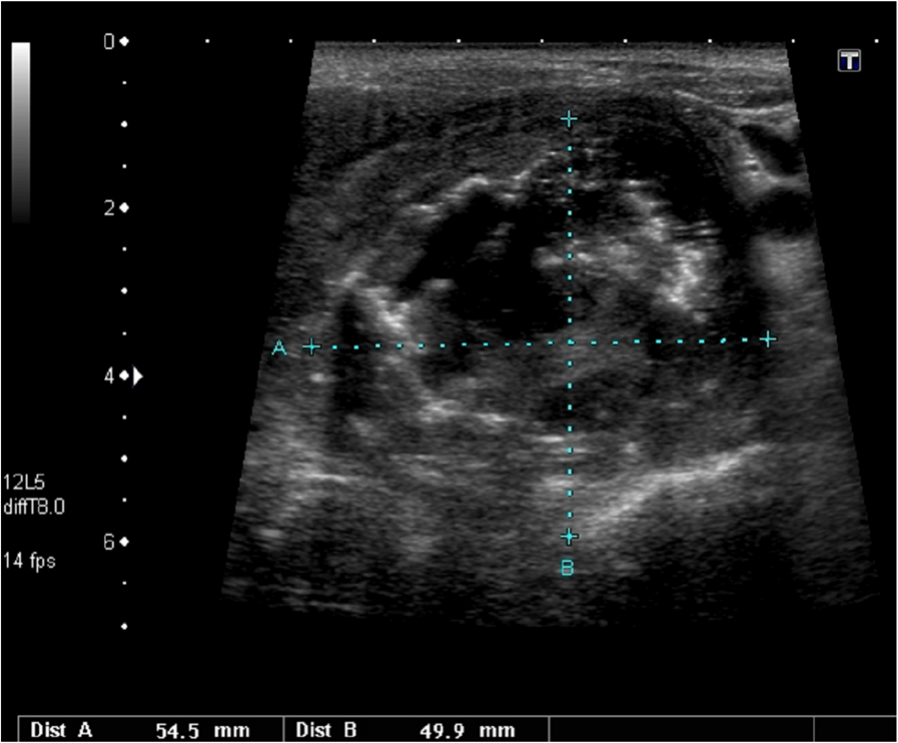
A large tumour of the thyroid, US pattern not typical for SAT; in the large hypoechoic mass the large abscess was visible; additionally, muscle infiltration and intra-tumour calcifications were clearly seen in the US

**Fig. 5:**
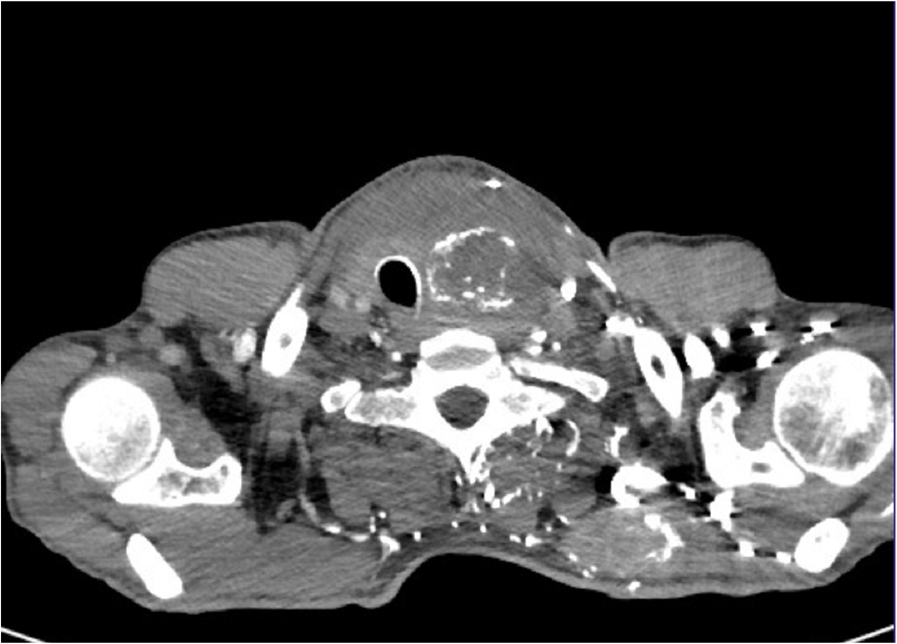
A large tumour of the thyroid, infiltrating surrounding structures, visible compression and displacement of the trachea – anaplastic thyroid cancer

A 45-year-old man, long time tobacco smoker, presented with two week history of marked thyroid pain and swelling. He also reported fever (up to 39 °C), weight loss and hoarseness. The patient’s GP initially diagnosed SAT based on the pain, fever and high ERS and started treatment with NSAID with initial pain relief. After a few days the severe pain relapsed despite treatment, and the patient was referred to hospital. In our Department his ESR was 93 mm/h and biochemical features of thyrotoxicosis were present, which may indeed strongly suggest SAT. In the neck, there was a hard, painful tumour, about 7 cm in size. The laboratory results obtained in our Department are presented in Table 1. US revealed slightly enlarged right thyroid lobe and significantly enlarged left lobe (65 × 57 × 101 mm) dominated by a heterogeneous tumour size of 55 × 50 × 72 mm, with numerous calcifications and a heterogeneous fluid space corresponding to abscess (Fig. 4). Tumour vascularisation was reduced. Almost the entire left lobe showed a faded structure of the parenchyma, with no evidently visible capsule, with features of infiltration of the neck structures. FNAB confirmed the presence of abscess, and after introduction of antibiotic therapy, the concentration of free thyroxine (FT4) gradually normalized. In the remaining FNAB material only blood and numerous granulocytes and macrophages were described. Neck CT revealed large tumour of the thyroid, with visible compression and displacement of the trachea and infiltration of the surrounding structures (Fig. 5). In chest CT, in both lungs, numerous milk-glass areas were present and the CT image corresponded to the diagnosis of *lympangiosis carcinomatosa*. The patient was referred to a surgery clinic, where only a small piece (20 mm) of the neck tumour was wedged to release the trachea; the complete surgery was impossible due to the extent of infiltration and hardness of the tumour. Histopathologically purulent necrosis was mostly observed, but together with clinical features, anaplastic thyroid cancer was suggested. The patient died a few months after diagnosis. Anaplastic thyroid cancer was confirmed.

### Case 3

**Fig. 6:**
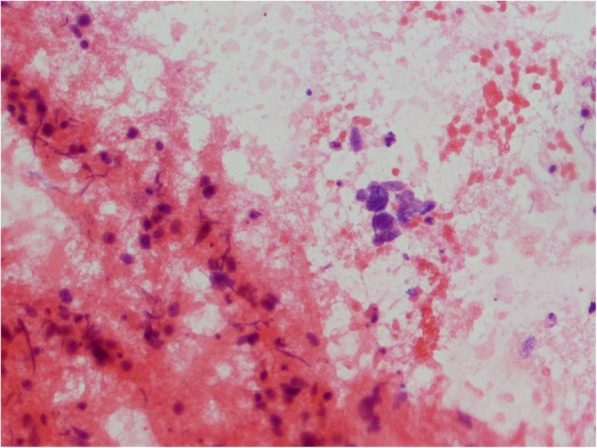
FNAB smear reveals groups, patches and shreds of tissues formed of anaplastic epithelial cells, partially spindle-shaped (light microscopy, hematoxylin-eosin staining; magnification × 500)

A 71-year-old woman, presented with a history of rapidly growing tender thyroid gland. Earlier, she was treated with radioiodine (^131^I) due to Graves` disease. Initially, the patient denied any other new symptoms, including fever and dyspnoea. She was consulted by her GP about two weeks before, and the initial diagnosis of SAT was made based on the hard painful thyroid tumour, increased CRP and low TSH. A treatment with NSAID was introduced and she was referred to our Department. Unfortunately, she did not report to the hospital immediately, but waited for two weeks for the treatment effect. In our Department her ESR was 94 mm/h and thyroid stimulating hormone (TSH) level was 0.02 mIU/l, which might have suggested SAT. In the neck, there was a hard, tender, huge goitre. The laboratory results obtained in our Department are presented in Table 1. US revealed huge nodular goitre with several hypoechoic nodules. On the right side of the neck pathological lymph nodes were visible, which raised a suspicion of malignancy. In all FNAB samples (right lobe, left lobe, lymph nodes) groups, patches and shreds of tissues formed of anaplastic epithelial cells, partially spindle-shaped, necrotic masses and granulocytes were described which confirmed diagnosis of anaplastic thyroid carcinoma (Fig. 6). At that time, she reported difficulty when swallowing. The patient was referred to a surgery clinic for palliative surgery. Total thyroidectomy was impossible to perform due to the infiltration of all adjacent structures including muscles, blood vessels, trachea and oesophagus. Only a part of the tumour was excised to release trachea and oesophagus. She died a few months later.

### Case 4

A 73-year-old woman presented with a history of enlarging tender thyroid gland. Several weeks earlier, in the outpatient clinic, she had laboratory features of hyperthyroidism and the treatment with methimazole (MMI) was started. Although SAT was suspected due to the typical symptoms and laboratory results, no glucocorticoid or NSAID therapy was introduced. Ultrasound examination was not performed. On admission to our Department, she had a hard, slightly tender nodular goitre in the neck. Her ESR was 39 mm/h, TSH level was 8.4 mIU/l on 10 mg of MMI daily. Other laboratory results obtained in our Department are presented in Table 1. US and CT revealed huge nodular goitre with two heterogeneous nodules in the left lobe, and one huge cyst in the right lobe. FNAB revealed numerous epithelial cells with marked anizocytosis, anizocariosis and macronucleosis, dispersed, in groups and lobes, and necrotic tissues. The description corresponds to anaplastic thyroid cancer. Pathological lymph nodes were present in CT image. The patient was referred to a surgery clinic, for palliative surgery. Total thyroidectomy was impossible to perform because of the extend of infiltration of adjacent structures. Unfortunately, detailed data regarding the surgical procedure are unavailable. The patient died a few months later. In this case the proper diagnosis was delayed significantly by incorrect initial diagnosis.

### Case 5

An 80-year-old woman presented with rapidly enlarging tender right thyroid lobe. In the neck, there was a hard, slightly tender nodule of the right lobe. Her ESR was 38 mm/h thus SAT was initially suspected in our Department. Other laboratory results obtained after admission are presented in Table 1. US revealed right lobe tumour 40 × 40 × 57 mm in size, hypoechoic with blurred margins and a few microcalcifications. Without those microcalcifications, the sonographic pattern of the tumour could be considered as SAT-typical, and the presence of microcalcifications was the first significant symptom of malignancy. FNAB revealed polymorphic cells with eosinophilic cytoplasm and hyperchromatic nuclei, with large nucleoli, dispersed, in groups and lobes, and numerous shreds of necrotic tissues. In the background of smears few lymphocytes and small groups of monomorphic cells of follicular origin were visible. The description corresponds to anaplastic thyroid cancer. The patient was referred to a surgery clinic for surgery. Unfortunately, the surgery occurred to be only palliative due to the excessive involvement of lymph nodes and adjacent structures. Further progression of the disease was very dynamic, and, despite the treatment, the patient died a few months later.

## Discussion and conclusions

Typical symptoms of subacute thyroiditis are neck pain and thyroid tenderness, which are accompanied by significantly elevated ESR. Pain can affect the whole thyroid or only one lobe, and the location of the pain can change over time. The occupied part of the thyroid gland is usually enlarged and hard in consistency. The inflammation causes destruction of thyroid follicles and a release of thyroid hormones into blood, which – in turn – results in elevation of free thyroid hormones and reduction of TSH level. Follicular cell damage causes an impaired transport of iodine, so RAIU is low in the affected parts of the thyroid.

However there are other aetiologies of painful goitre, including primary fast growing thyroid neoplasms or thyroid metastases [7–10]. Rapid enlargement of the thyroid causes painful stretching of the gland capsule. Very aggressive tumours quickly infiltrate surrounding tissues, including neck muscles, which also causes pain with exactly the same radiation as in SAT. These tumours, similarly to the inflammation in SAT, are hard in consistency. Rapidly growing primary thyroid carcinomas, including mainly very poorly prognostic anaplastic cancer, are characterized by a fulminant course of the disease, and delayed diagnosis - even a week or two - can dramatically reduce the patient’s life expectancy and deteriorate life quality. Thyroid metastases can also grow rapidly, stretching the capsule, and infiltrate surrounding tissues, which result in the same neck pain [11–17]. Thyroid abscess is difficult to distinguish from SAT [7] and one should remember that thyroid abscess is a very rare condition which may be the result of infection of decaying necrotic tissues in anaplastic thyroid cancer, as it occurred in Patient No. 2. All of the above causes of painful goitre are accompanied by significantly increased ESR resulting from progressive cancer disease. ESR in the thyroid abscess is always very high, regardless of whether it is accompanied by cancer or occurs as a separate disease entity [7]. Even the accompanying biochemical hyperthyroidism cannot be the basis for the exclusion of malignant tumour, because both inflammation and rapidly progressive cancer infiltration can cause destruction of thyroid follicles and the release of hormones, as it was in Patients No. 2, 3 and 4.

To our knowledge, this is the largest group of patients with thyroid anaplastic cancers mimicking SAT, described so far.

The problem of distinguishing the causes of painful goitre with accompanying other features of SAT is scarcely discussed in the literature. Meier and Nagle [7] reported 49-year-old men with severe pain of enlarged left thyroid lobe, which was firm and tender. Lab results revealed ERS 50 mm/h and features of thyrotoxicosis. f The diagnosis of SAT was made and the patient was treated with prednisone in high doses with initial clinical improvement. However, after 10 days his pain worsened and FNAB was performed revealing purulent material The large painful abscess appeared to arise from an extension of laryngeal carcinoma [7]. This case, as well as our Patient No. 2 presented above, are important examples that thyroid abscess in an adult should always trigger diagnostic alertness, as it is often the first symptom of an advanced cancer disease. It should also be emphasized that neck abscess is extremely difficult to distinguish from SAT based only on clinical symptoms and ESR, thus US is mandatory in all cases.

The cases presented in the paper clearly indicate that primary thyroid tumours with poor prognosis, in particular anaplastic cancer, may be clinically similar to SAT. Typical symptoms include neck pain, radiating to the ear, mandible and/or chest with increased ESR. Differentiated thyroid cancers progress much slower than anaplastic cancers, rarely give extensive infiltration of neck tissues and usually are not associated with significant ESR. However, Prakash et al. [8] described a case of follicular thyroid cancer that manifested as hard, tender right thyroid lobe enlargement, associated with modest elevation of thyroid hormone levels and suppressed thyroid RAIU. Unfortunately, no ESR result of this patient is available in that paper. In such cases, pain is caused by rapid tumour growth which is, however, not characteristic for the follicular thyroid cancer.

SAT-like symptoms may be caused by a non-thyroid cancer that infiltrates the thyroid or metastasizes to the thyroid gland. An example of neoplastic extension to the thyroid was presented by Meier and Nagle [7], who reported 19-year-old man with neck pain and swelling. On examination, there was firm and tender 4 cm mass in the region of right thyroid lobe and isthmus. White blood count (WBC) and ESR were increased. FNAB procedure was painful and did not provide adequate material. A tentative diagnosis of SAT was made and the patient received initial dose 60 mg of prednisone with a definite improvement. After a month of tapering the dose the thyroid mass was still hard. Repeated FNAB revealed Hodgkin’s disease. CT images showed 7 cm mediastinal mass which extended into right thyroid lobe [7]. In this case, the initial SAT diagnosis significantly delayed proper treatment, similarly to most of our patients presented above. Most cases of thyroid lymphomas are non-Hodgkin’s ones. Hamburger reported a few cases of painful thyroid lymphomas, which may be difficult to differentiate from SAT on the basis of pain and ESR [9]. Gochu at al. [10] described a HIV positive patient who presented with diffusely enlarged, firm and extremely tender thyroid gland, tachycardia and tremor of the fingers suggested hyperthyroid state. Laboratory features of thyrotoxicosis were present and ESR was 45 mm/h. All these findings were suggestive of SAT. Fortunately, further diagnostics was carried out. Two FNABs revealed only necrotic material and were non-diagnostic. An open biopsy demonstrated malignant lymphoma (diffuse large cell type). Chemotherapy with bleomycin, cyclophosphamide, doxorubicin, vincristine and dexamethasone was introduced with a dramatic reduction of the goitre and rapid normalization of thyroid tests [10].

Several cancers metastasize to the thyroid. Based on recent literature, the most common malignancies that metastasize to the thyroid gland are renal cell (48.1%), colorectal (10.4%), lung (8.3%), breast carcinoma (7.8%), and sarcoma (4.0%) [11]. Metastases to the thyroid are more common in women than men (female to male ratio = 1.4). In most cases the diagnosis of primary cancer is made many months before the diagnosis of thyroid metastases. In about 20% of cases the diagnosis of primary tumour and its metastases to the thyroid is synchronous [11]. In very rare cases, such as in our Patient No. 1, metastasis is the first symptom of an advanced cancer. Patient No. 1 reported symptoms highly suggestive of SAT, which resulted in 3-week delay of diagnosis. In the case of NSCLC metastases, even the slightest delay in the diagnosis is associated with a dramatic deterioration of prognosis and shortening of survival. Thyroid is an extremely rare site of NSCLC metastases. The most common sites are brain, bone, liver, adrenal glands, contralateral lung and distant lymph nodes. Other sites are defined as uncommon as they cover less than 5% of all metastases [12]. Niu et al. [12] reported that NSCLC thyroid metastases cover only 0.07% of all metastases of this cancer. Our Patient No. 1 is one of very few cases reported so far. Similar case was described only by Boukir et al. [13] who reported 51 year old woman with hard, well-defined, painful swelling in the left lobe which also occurred to be NSCLC metastasis.

Thyroid metastases from lung cancers other than NSCLC are much more common. Can and Köksal [14] reported 55-year old man with diagnosed small cell lung carcinoma (SCLC) and right thyroid lobe nodule, which was hypoechogenic and had blurred irregular borders in US, similarly to SAT. FNAB confirmed small cell carcinoma metastasis [14]. Several similar cases of SCLC thyroid metastases have been reported so far [15, 16] but most of them were not initially suggestive of SAT, due to lack of pain and no ESR result available. Elevated thyroid hormones and low TSH levels are often present [14]. All those patients with SCLC were long time heavy smokers. Other lung cancers can also metastasize to the thyroid and give SAT-like symptoms. Meier and Nagle [7] reported a 54-year old man presented with 5-week history of painful goitre, dyspnoea and dysphagia. As SAT was suspected, he received prednisone with improvement. When prednisone was stopped the pain recurred, so the treatment was re-instituted. Thyroid was diffusely enlarged and tender. FNAB revealed cancer cells and the final diagnosis of metastatic large-cell pulmonary adenocarcinoma was made [7]. There is no available data concerning smoking history of this patient. But most of lung adenocarcinoma patients are smokers. Dao et al. [17] recently reported a 59-year old man with lung adenocarcinoma and very fast diagnosis of thyroid metastasis, despite diagnostic difficulties. In this case, repeated FNAB of the suspected thyroid nodule were negative and total thyroidectomy with neck dissection was performed allowing finally to confirm diagnosis of lung cancer metastasis [17]. The diagnostic difficulties in this case are similar to our Patient No. 2, in whom FNAB did not reveal cancer cells. In our patient complete surgery was impossible due to the extent of infiltration and hardness of the tumour, but partial operation let us to make diagnosis of anaplastic thyroid cancer, reduce the pressure on the trachea and improve breathing. Patients with non-diagnostic FNAB of lesions highly suspicious of malignancy should always be referred to urgent surgery, as proper diagnosis allows to choose the best possible treatment which can significantly extend life expectancy.

Symptoms of thyroid amyloidosis may be similar to SAT, with painful goitre, high ESR, low RAIU and good response to glucocorticoids. There are, however, other features of systemic amyloidosis, which exclude SAT diagnosis. Ikenoue et al. [18] reported two men with SAT-like symptoms including tender diffused firm goitres, low RAIU, very high ESR and rapid response to glucocorticoids. Both patients had history of diarrhoea and abdominal pain, both were undernourished. In both cases the initial diagnosis was SAT as FNAB was not performed at that time. Because of the persistence of goitre after pain remission, repeated recurrence of symptoms with always the same thyroid localization and presence of gastrointestinal and – later – renal abnormalities, thyroid biopsy was performed and revealed amyloid deposition [18]. It should always be remembered, that differential diagnosis of symptoms suggestive of SAT should also include amyloidosis.

Painful goitre may also results from other diseases such as haemorrhage, infarction of thyroid nodule, *Pneumocystis carinii* infection and sometimes chronic thyroiditis or Graves` disease. However, these conditions are not associated with increased ESR and such inflammation markers as C-reactive protein or WBC, and are usually easy to differentiate from SAT. And most importantly, in these cases a slight delay in the diagnosis does not constitute a direct threat to the patient’s life.

The only method of distinguishing cancerous and SAT lesions in the described patients was neck US, in which the image of the thyroid cancer is usually different from the typical image of diffused hypoechoic areas in SAT. Therefore, US examination should be obligatory in every patient with suspected SAT. However, some features of the US image of malignant lesions may be similar to SAT, such as blurred lesion margins, hypoechogenity, or significantly reduced vascularisation. The same observation was presented by Pan et al. [19] who evaluated sonographic features for distinguishing SAT from malignant thyroid nodules. They concluded that useful sonographic predictors of SAT are poorly defined margin, centripetal reduction of echogenicity and the absence of internal vascularity. However a considerable overlap of features of malignant nodules and SAT was observed [19]. The decisive test should therefore be FNAB, especially when there are diagnostic doubts, and optimally in all patients with suspected SAT. In the opinion of the authors, the purpose of the FNAB is not to necessarily obtain a typical cytological image of SAT to confirm diagnosis, because it is not possible in every patient with SAT. The priority goal of FNAB should be to exclude malignancy that mimics SAT. Obtaining FNAB result typical for SAT provides final confirmation, but the absence of multinucleated macrophages does not exclude diagnosis if other criteria are met. If FNAB samples are non-diagnostic in a highly suspicious thyroid lesion, patient should be referred to thyroid surgery to make a proper diagnosis and to introduced adequate treatment. As it was presented in our cases and in the review of the literature, good response to glucocorticoids cannot serve as a marker of SAT, because the symptoms improvement was observed in most cases, even in thyroid cancer or metastasis.

Based on our own experience and literature review, the authors suggest that thyroid gland US should be necessarily included in the main criteria of SAT diagnosis, together with FNAB result excluding the presence of malignant tumour, if only it is possible to perform a diagnostic FNAB. If FNAB does not provide a diagnostic result, and there are even slight suspicious of malignancy, thyroid surgery should be considered to obtain definitive diagnosis, because only the correct diagnosis allows to extend the life expectancy of a patient with a malignant tumour of poor prognosis. One should always remember that thyroid primary or metastatic cancer can clinically mimic SAT, so SAT diagnosis can be made only after exclusion of malignancy.

Taking into account the above observations, we propose the following SAT recognition criteria:

- Elevation of ESR or at least C-reactive protein.
- Hypoechoic area/areas with blurred margin and decreased vascularisation in US.
- FNAB confirmation of SAT or at least FNAB exclusion of malignancy.
- Hard thyroid swelling.
- Pain and tenderness of the thyroid gland/lobe.
- Elevation of serum FT4 and suppression of TSH.
- Decreased RAIU.

To undoubtedly diagnose SAT the first 3 criteria should be met, together with at least one other criterion.

## Data Availability

All data generated or analysed during this study are included in this published article.

## Abbreviations

anti-Tg: thyroglobulin antibodies
anti-TPO: thyroid peroxidase antibodies
CRP: C reactive protein
CT: Computed tomography
ESR: Erythrocyte sedimentation rate
FNAB: Fine needle aspiration biopsy
FT3: Free triiodothyronine
FT4: Free thyroxine
MMI: Methimazole
SAT: Subacute thyroiditis
TRAb: TSH receptor antibodies
TSH: Thyroid stimulating hormone
US: Ultrasound
WBC: White blood count
